# Does a reduction in alcohol use by Dutch high school students relate to higher use of tobacco and cannabis?

**DOI:** 10.1186/s12889-015-2149-8

**Published:** 2015-08-26

**Authors:** Claudia E. Verhagen, Daan G. Uitenbroek, Emilie J. Schreuders, Sabah El Messaoudi, Marlou L. A. de Kroon

**Affiliations:** Department of Epidemiology & Health Promotion, Public Health Service of Amsterdam, P.O. Box 2200, 1000 CE Amsterdam, The Netherlands; Department of Public Health, Erasmus Medical Centre, Rotterdam, The Netherlands

## Abstract

**Background:**

Substance use of adolescents was investigated in a region around Amsterdam, the Netherlands, in the period 2005–2009. The study was intended to find out to what extent behaviour related to different substances are interrelated and how trends develop in different subgroups.

**Methods:**

Two cross-sectional surveys were conducted among Dutch students in the second and fourth year of secondary school, aged 13-16 [*n* = 1,854 in 2005; *n* = 2,088 in 2009] by making use of an online questionnaire including questions about alcohol consumption, tobacco use (smoking behaviour) and cannabis use. Two educational levels were included.

**Results:**

Decreases in alcohol consumption, tobacco and cannabis use were found between 2005 and 2009. The strongest decline was seen in alcohol consumption. Last month drinking decreased from 61.8 % in 2005 to 36.5 % in 2009. Last month binge drinking decreased from 38.7 % in 2005 to 24.0 % in 2009. Reduced alcohol consumption was found among boys and girls, for all ages and in both educational levels. Changes were strongest among 13-year-olds. Weekly or daily smoking declined between 2005 and 2009 among 13-year-olds, girls and students in the lower schooling level. Last month cannabis use decreased among girls and students in the higher schooling level. In both 2005 and 2009 clustering with alcohol consumption was found for the use of other substances.

**Conclusions:**

Between 2005 and 2009 alcohol consumption strongly decreased among high school students. This may be due to the national prevention campaign which in the same period highlighted the importance of not drinking before the age of 16. The decrease in smoking and cannabis use between 2005 and 2009 may be due to clustering with alcohol consumption. A reduction in the use of alcohol in adolescence did not lead to replacement by tobacco or cannabis use.

## Background

The impact of substance use on our society is reflected by its harmful effects. Smoking causes about 5 million deaths annually worldwide and the harmful use of alcohol results in approximately 2.5 million deaths each year [1, 2]. Among adolescents, risk behaviours such as smoking, alcohol and drug use are found to cluster with other risk behaviour such as unprotected sexual intercourse and antisocial or criminal behaviour [3–6]. These (clustered) risk behaviours are associated with poor school results [7–9], and an increased risk of future morbidity and premature mortality [1, 2, 10, 11]. Starting to use alcohol, tobacco or drugs at a young age is a risk factor for the use or abuse as an adult [12–16]. Prevention of substance use in young adolescents is therefore crucial in public health.

In 2003 Dutch adolescents were among Europe’s highest alcohol consumers [17]. Therefore, the political and social attention for harmful alcohol use among young people in the Netherlands has increased considerably. Since 2006, national drinking policies and school-based intervention programmes target adolescents and their parents with the message to postpone drinking until the age of 16 [18]. Other policies in the Netherlands aim at discouraging and postponing tobacco smoking by introducing an age limit [age 16] on buying tobacco products, by prohibiting advertising and by introducing smoking bans in public places [19]. Policies on marijuana use, however, have not changed much and health promotion is relatively muted [20]. Drug policy in the Netherlands mainly aims at controlling and reducing drug-related problems and has done so since its development in the mid-seventies of the previous century [21].

Recent national population studies showed promising trends on substance use among adolescents in the Netherlands. Between 2003 and 2009 lifetime and present (last month’s) use of alcohol among Dutch adolescents strongly decreased among those under 16 [22, 23]. Binge drinking among adolescents decreased between 2005 and 2007 [23]. Indeed, several studies support the idea that interventions addressing adolescents as well as their parents have been effective in preventing alcohol use in early adolescence [24–27]. Between 2001 and 2009 decreasing trends were also found in smoking (lifetime and daily) and cannabis (last year) use by adolescents in the Netherlands [23, 28].

Although the Dutch policies appear to be successful, the coherence in these policies and their evaluation is lacking; there is an alcohol policy, a tobacco policy, and a drugs policy. Policies are rarely developed and evaluated on a more general level. To date, there are no studies about whether a decrease in the use of one substance leads to an increase in the use of another. In other words, is there replacement? Does a reduction in the use of alcohol lead to more drug use? Do people who stopped smoking drink more in compensation? Personality characteristics and the developmental stage of adolescents are related to a need for risk or sensation seeking behaviour exercised in the form of different risky health behaviours dependent on opportunity [29]. Therefore, in this paper, the emphasis is on the question to what extent behaviour and time trends in the use of different substances are interrelated. In this context we are furthermore curious to find out how trends develop in different subgroups. Does differential changes in different subgroups, as was also shown by others [27, 30], lead to different replacements? Previous studies have shown that health promotion is effective in different ways and might need to target different aspects in different demographic groups [31]. Therefore, we studied substance use by gender, age and educational level to get insight in possible effects of the campaigns. With this study we aim to provide points of reference for national policies or campaigns in other countries in or outside Europe.

## Methods

### Setting and study population

The Public Health Service of Amsterdam, the Netherlands, organizes general health surveys in a region around Amsterdam (the Amstelland region) among high school students (secondary education). Two studies, in 2005 and 2009, provided the possibility to monitor substance use among adolescents in this region. The Amstelland region consists of four municipalities (Aalsmeer, Uithoorn, Ouder-Amstel and Amstelveen) with about 145,000 inhabitants. Out of a total of nine high schools in the Amstelland region eight were included in the surveys. One school was unable to participate in the study due to lack of time. Second and fourth year students, aged 13, 14, 15 or 16, living in the Amstelland region were asked to complete an online questionnaire in a classroom setting. Participation was anonymous and voluntary. Students were divided into two schooling levels; VMBO students (lower educational level) and HAVO/VWO students (higher educational level). Seven participating schools included the entire second and fourth year, one school only the fourth year. The study population in 2005 consisted of 1,854 students and in 2009 of 2,088 students. For 66 classes in 2005 (1,564 students) and 56 classes in 2009 (1,324 students) participation was followed. The response was 91.4 % in 2005 and 92.5 % in 2009; the main reason for non-response was illness of the student.

### Questionnaires

The questionnaires in 2005 and 2009 included questions about demographic and baseline characteristics (such as age, gender, educational level and ethnicity) and several health-related subjects including questions about alcohol consumption, smoking behaviour and cannabis use. In both surveys, of 2005 and 2009, the same questions were used to investigate drug using habits. The eight questions to assess alcohol-drinking habits were also similar in both surveys except for the possible answers to the first question “What kind of alcohol do you drink?”. The answer possibilities in 2005 and 2009 differed in order to assess whether a student had ever drunk alcohol. In 2005 the possible answer was “I don’t drink alcohol at all” and in 2009 “I have never drunk alcohol”. Both answers excluded the students from further questions about alcohol use. In the survey of 2005 smoking habits were investigated using one question. In 2009 this question was split up into two questions with the same answer options.

### Statistical analyses

The outcome variables were last month alcohol-drinking, last month binge drinking, last month being drunk or tipsy, weekly and daily smoking and lifetime cannabis use and last month cannabis use. Explanatory variables were gender, age and educational level.

Differences in baseline characteristics (age, gender, educational level and ethnicity) between the study population in 2005 and 2009 were examined using Chi-squared tests.

For comparing the surveys (2005 and 2009) logistic regression analyses were applied to correct for differences between the surveys by gender, age and educational level. For the subgroup analyses by gender, by age and by educational level respectively the analyses were corrected by including the two remaining variables to the model. The regression coefficients for the years of investigation were used to calculate new ‘adjusted’ prevalence rates in percentages for 2009 using the SISA’s logit module [32]. To study differences between the two surveys (2005 and 2009) for the subgroups by educational level, by age and by gender, 2^nd^ order interaction terms of these variables with survey year were added to the regression models. Analyses were performed with SPSS version 19.

### Ethical approval and consent

This study has been reported to the Dutch data protection authority and meets national ethics and privacy requirements. Parents were informed of the data collection by mail and they could refuse entry of their child into the data collection. This method of passive agreement is in accordance with Dutch legal standards [33, 34].

## Results

Differences in baseline characteristics (age, gender, educational level and ethnicity) are presented in Table 1. With respect to age distribution a significant difference was found between the populations in 2005 and 2009. These differences are considered in the analysis by using multivariate logistic regression.

**Table 1:** Characteristics of the study population in the survey of 2005 and 2009

	2005 *n* (%)	2009 *n* (%)
	*N* = 1,854	*N* = 2,088
*Gender*		
Boys	958 (51.7)	1,023 (49.0)
Girls	896 (48.3)	1,065 (51.0)
*Age (years)* ^a^		
13	654 (35.3)	622 (29.8)
14	342 (18.4)	316 (15.1)
15	525 (28.3)	753 (36.1)
16	333 (18.0)	397 (19.0)
*Level of education*		
Low VMBO	773 (41.7)	825 (39.5)
High HAVO/VWO	1081 (58.3)	1,262 (60.5)
*Ethnicity*		
Western	1,661 (89.7)	1,872 (89.7)
Non-western	191 (10.3)	215 (10.3)

^a^compared with Chi-squared test (*p* <0.05)

Table 2 shows the prevalence rates for alcohol use, smoking and cannabis use among 1,854 high school students in 2005 and the prevalence rates among 2,088 high school students in 2009, adjusted for age, gender and educational level. There is a discernible decrease in alcohol consumption, smoking and cannabis use in the period 2005 - 2009. The strongest decline over time was found for alcohol use. The prevalence of last month (current) drinking decreased from 61.8 % in 2005 to 36.5 % in 2009. For last month binge drinking and last month being drunk or tipsy the prevalence decreased from 38.7 % in 2005 to 24.0 % in 2009 and from 29.7 % in 2005 to 16.5 % in 2009, respectively. Decreases were seen between 2005 and 2009 for weekly smoking and for lifetime and last month cannabis use as well.

**Table 2:** Prevalence of alcohol use, smoking and cannabis use among students (aged 13–16) in 2005 and 2009 by gender (%)

	Total		Boys		Girls	
	2005	2009^b^	2005	2009^c^	2005	2009^c^
	*N* = 1,854	*N* = 2,088	*N* = 959	*N* = 1,023	*N* = 895	*n* = 1,064
*Alcohol*						
Last month	61.8	36.5^a^	60.5	37.7^a^	63.1	35.1^a^
Last month binge drinking^d^	38.7	24.0^a^	39.9	27.5^a^	38.0	20.9^a^
Last month been drunk or tipsy	29.7	16.5^a^	28.8	19.3^a^	30.7	13.9^a^
*Smoking*						
Weekly	13.9	11.4^a^	12.0	11.4	16.0	10.4^a^
Daily	9.7	7.9	9.0	8.8	10.5	7.2^a^
*Cannabis*						
Lifetime prevalence	21.6	15.3^a^	23.2	21.0	19.9	9.9^a^
Last month	9.9	7.6^a^	11.0	10.7	8.7	4.1^a^

^a^significant difference between the 2005 and 2009 survey: *p* <0.05, ^b^adjusted for gender, age and educational level, ^c^adjusted for age and educational level, ^d^binge drinking: five or more glasses of alcohol per occasion

Similar decreases between 2005 and 2009 in prevalence rates of alcohol consumption (current and binge drinking) were found among boys and girls (Table 2). The decline of being drunk or tipsy was stronger among girls than among boys (*p* = 0.006). Moreover, the decrease of smoking and cannabis use was found only among girls.

These trends have resulted in significant differences in prevalence rates of substance use between boys and girls in 2009 that were not found in 2005. In 2009 boys were more often than girls binge drinkers last month (OR 1.33 95 % CI 1.07–1.64) and were more often drunk or tipsy (OR 1.35 95 % CI 1.07–1.72). Moreover, in 2009 more boys than girls reported to have used cannabis at least once (OR 2.34 95 % CI 1.82–3.02) and to use cannabis weekly (OR 2.90 95 % CI 2.04–4.14). In 2009 smoking habits in boys and girls were similar, whereas in 2005 girls reported more weekly smoking than boys (OR 0.65 95 % CI 0.49–0.86).

Table 3 shows the age-related prevalence of alcohol consumption, smoking and cannabis use among high school students aged 13, 14, 15 and 16 in both surveys (2005 and 2009). For all ages a decrease in the prevalence of alcohol consumption was found between 2005 and 2009. The strongest decrease was found among 13-year-old students for last month drinking, last month binge drinking and last month being drunk or tipsy. The smallest decrease was found among 16-year-olds. In smoking habits the only significant decrease was found for 13 years olds between 2005 and 2009. On the other hand, the proportion of students who had used cannabis at least once decreased between 2005 and 2009 among students older than 13: among 14-year-olds from 15.9 % to 9.8 %, among 15 year olds from 31.6 % to 21.9 %, and among 16-year-olds from 41.9 % in 2005 to 34.3 % in 2009.

**Table 3:** Prevalence of alcohol use, smoking and cannabis use among students (aged 13–16) in 2005 and 2009 by age and by educational level (%)

			Age						Educational level		High HAVO/VWO	
		13	14		15		16		Low VMBO			
	2005	2009^b^	2005	2009^b^	2005	2009^b^	2005	2009^b^	2005	2009^c^	2005	2009^c^
	*N* = 655	*N* = 662	*N* = 341	*N* = 315	*N* = 525	*N* = 753	*N* = 333	*N* = 397	*N* = 773	*N* = 824	*N* = 1,081	*N* = 1,261
*Alcohol*												
Last month	42.9	14.6^a^	51.0	29.4^a^	78.5	58.5^a^	83.5	76.4^a^	64.2	43.2^a^	60.1	32.0^a^
Last month binge drinking^d^	17.8	7.3^a^	30.6	19.2^a^	53.0	35.1^a^	65.5	54.9^a^	44.5	34.2^a^	34.5	17.4^a^
Last month been drunk or tipsy	10.5	2.8^a^	19.3	9.3^a^	44.8	26.6^a^	54.3	42.2^a^	32.2	22.6^a^	28.0	12.3^a^
*Smoking*												
Weekly	6.4	3.2^a^	12.0	10.6	16.8	13.0	26.1	26.3	21.9	17.6^a^	8.2	7.1
Daily	4.1	2.1^a^	8.8	7.2	11.8	8.9	18.3	18.8	16.2	12.6^a^	5.1	4.7
*Cannabis*												
Lifetime prevalence	6.3	4.4	15.9	9.8^a^	31.6	21.9^a^	41.9	34.3^a^	25.6	19.1^a^	18.7	12.3^a^
Last month	3.3	1.8	6.9	6.1	12.8	10.2	21.5	16.0	11.0	10.6	9.1	5.4^a^

^a^significant difference between the 2005 and 2009 survey: *p* <0.05, ^b^adjusted for gender and educational level, ^c^adjusted for gender and age, ^d^binge drinking: five or more glasses of alcohol per occasion

These trends have resulted in an increased age-related difference in the prevalence of alcohol consumption and smoking between 13 and 16-year-olds. Moreover, the proportion of binge drinking students (last month) among the alcohol-drinking students (last month) decreased from 61.0 % in 2005 to 55.1 % in 2009 (*p* = 0.012). This decrease is due to the decrease in the proportion of 15- and 16-year-old binge drinking students from 67.2 % and 78.4 % in 2005 to 57.6 % and 70.1 % in 2009, respectively.

In both educational levels the alcohol use between 2005 and 2009 decreased (Table 3). The percentage of VMBO students (lower educational level) that were current drinkers decreased from 64.2 % in 2005 to 43.2 % in 2009. Among HAVO/VWO students (higher educational level) 60.1 % were current drinkers in 2005, whereas in 2009 this percentage was 32.0 %. The prevalence of current binge drinking decreased between 2005 and 2009 among both VMBO students and HAVO/VWO students. The prevalence decreases in current binge drinking and being drunk/tipsy were larger among HAVO/VWO students than among VMBO students (*p* = 0.007 and *p* = 0.016, respectively).

A time trend in smoking habits was only observed in VMBO students. Between the surveys in 2005 and 2009 weekly smoking among VMBO students declined from 21.9 % in 2005 to 17.6 % in 2009, and daily smoking from 16.2 % to 12.6 %. In 2005, the prevalence of smoking among HAVO/VWO students was already three times lower than among VMBO students. Between 2005 and 2009 lifetime cannabis use decreased in both educational levels. Last month cannabis use only decreased among HAVO/VWO students.

In 2009 students with a lower educational level were still more at risk of using stimulants than students with a higher educational level. VMBO students were more often last month drinkers (OR 1.50 95 % CI 1.21–1.86), last month binge drinkers (OR 2.29 95 % CI 1.83–2.88), last month being drunk or tipsy (OR 1.83 95 % CI 1.42–2.35), weekly smokers (OR 2.62 95 % CI 1.96–3.50), daily smokers (OR 2.74 95 % CI 1.95–3.87), weekly (OR 1.55 95 % 1.20–2.02) and ever cannabis users (OR 1.83 95 % CI 1.30–2.59) than HAVO/VWO students.

### Clustering of substance use

We investigated the clustering of substance use among the high school students in both the 2005 and 2009 survey (Table 4). Students who did not drink alcohol in the last month hardly used any other substances. Multivariate logistic analysis with correction for gender, age and educational level showed that the prevalence rate of students who did not use substances (no recent alcohol, no weekly smoking, no cannabis use ever) increased from 36.6 % in 2005 to 60.7 % in 2009 (*p* = <0.001).

**Table 4:** Clustering of substance use: percentages of students (aged 13–16) using alcohol in the last month and/or smoking last week and/or using cannabis ever in the survey of 2005 and 2009 (%)

	2005	2009
	(*N* = 1,850)	(*N* = 2,044)
		^a^
No alcohol, no smoking, no cannabis	36.6	52.6
No alcohol, smoking, no cannabis	0.5	0.5
No alcohol, no smoking, cannabis	0.6	1.7
No alcohol, smoking, cannabis	0.5	0.9
Alcohol, no smoking, no cannabis	38.8	27.2
Alcohol, smoking, no cannabis	2.5	2.6
Alcohol, no smoking, cannabis	10.1	6.6
Alcohol, smoking, cannabis	10.4	7.9

^a^compared with Chi-squared test; *p* <0.05

Multivariate logistic regression analysis with correction for gender, age and educational level, showed that in 2009 students who had recently drunk alcohol were about eight times more at risk to smoke weekly and/or to use cannabis (ever) than students who did not drink any alcohol in the last month (OR 7.59 95 % CI 5.56–10.38), in 2005 the risk was about ten times higher (OR 9.61 95 % CI 6.47–14.28). We investigated clustering of substance use in different subgroups and whether it changed over time. Table 5 shows the use of tobacco (weekly) and/or cannabis (ever) among alcohol-drinking and non-alcohol-drinking students in 2005 and in 2009, in relation to gender, age and educational level. Only among girls the clustered use of alcohol, tobacco and cannabis differed between 2005 and 2009. In the period 2005–2009 the prevalence of the use of tobacco and/or cannabis among alcohol-drinking girls had decreased from 35.3 % to 27.1 % (*p* = <0.001).

**Table 5:** Prevalence of smoking last week (S) and/or cannabis use at some time (C) among alcohol-drinking and non-alcohol-drinking (last month) students in the survey of 2005 and 2009 in relation with gender, age and educational level (%)

	Non-alcohol-drinking students				Alcohol-drinking students			
	No S, No C		S and/or C		No S, No C		S and/or C	
	2005	2009	2005	2009	2005	2009	2005	2009
	*N* = 677	*N* = 1,075	*N* = 30	*N* = 62	*N* = 718	*N* = 556	*N* = 425	*N* = 351
*Total*	95.8	94.8^b^	4.2	5.2^b^	62.8	65.2^b^	37.2	34.8^b^
*Gender*								
Boys	95.5	92.9^c^	4.5	7.1^c^	61.0	57.1^c^	39.0	42.9^c^
Girls	96.0	97.0^c^	4.0	3.0^c^	64.7	72.9^a c^	35.3	27.1^a c^
*Age (years)*								
13	98.9	97.5^d^	1.1	2.5^d^	81.4	77.4^d^	18.6	22.6^d^
14	94.0	95.0^d^	6.0	5.0^d^	69.0	66.3^d^	31.0	33.7^d^
15	90.3	91.0^d^	9.7	9.0^d^	58.0	63.4^d^	42.0	36.6^d^
16	90.9	85.5^d^	9.1	14.5^d^	47.3	51.2^d^	52.7	48.8^d^
*Level of education*								
Low VMBO	93.1	93.0^e^	6.9	7.0^e^	55.2	58.3^e^	44.8	41.7^e^
High HAVO/VWO	97.4	96.2^e^	2.6	3.8^e^	68.7	71.0^e^	31.3	29.0^e^

^a^significant difference between the 2005 and 2009 survey: *p* <0.05

^b^adjusted for gender, age and educational level

^c^adjusted for age and educational level

^d^adjusted for gender and educational level

^e^adjusted for age and gender

## Discussion

In this study we looked at the following questions: What are the trends in Dutch adolescents’ (aged 13–16) alcohol consumption, smoking behaviour and cannabis use between 2005 and 2009, in what groups are changes concentrated, and how is substance use clustered?

Our main finding is that in a period of only four years (2005–2009) a significant change in the prevalence of substance use among high school students has occurred among 13- to 16-year-old students with regard to all surveyed behaviours. Strongest decreases were found for alcohol use in last month, last month binge drinking and last month being drunk or tipsy and were found most prominent among 13-year-old students. These decreases were less with increase of age but were still significant among 16-year-olds. Similar reductions in alcohol consumption were found among boys and girls. In both educational levels the alcohol use between 2005 and 2009 decreased. The decreases in prevalence found for current binge drinking and being drunk or tipsy were stronger among students with a higher educational level. A significant decrease in cannabis use in the last month was found among girls and students with a high educational level. The decrease of lifetime cannabis use was significantly stronger among girls than boys. This may be explained by the relatively high cannabis use among the girls in 2005. In this study the decrease in smoking behaviour was significant among 13-year-old students, girls and students with a lower schooling level. Limitations to the interpretation of our results in being representative for the Dutch school-going population of 13- to 16-year-olds are (1) inclusion of only second and fourth year students in secondary school (2) the fact that in the study population we included more students in the higher than in the lower educational level compared with the overall high/low educational level ratio in the Netherlands, which was 46 %/54 % in 2005 and 50 %/50 % in 2009 [35].

Other Dutch study results are in line with our results; between 2003 and 2011 the lifetime and actual (last month) alcohol use strongly decreased among Dutch adolescents under 16: binge drinking decreased as well between 2003 and 2011 [17, 22, 23, 27, 35]. The decrease in last month drunkenness we found in our study was not shown in national studies [36]. Equally so, the decrease in cannabis use among girls in this study was not found in national studies and may therefore be a local characteristic [36]. Other Dutch studies did show decreasing trends in cannabis use (last year) among adolescents in the Netherlands between 2001 and 2009 [23]. Decreasing trends between 2001 and 2009 with regard to smoking [lifetime and daily] were also found in the national studies [23, 28]. Interestingly, we found that in 2005 smoking was still more common among girls, whereas in 2009 the gender-gap was no longer statistically significant. The latter was also found in other Dutch studies, and in several other western European countries [22, 23, 37].

To put those finding of decreasing trends of substance use in the Netherlands in perspective with other countries, the European School Survey Project on Alcohol and Drugs (ESPAD) reports are the best way to do this. ESPAD is carried out in 35 European countries, including 15- and 16-year-old students, with surveys in 1995, 1999, 2003, 2007 and 2011. ESPAD trend analyses between 2003 and 2011 showed that in the majority of countries last month alcohol use was unchanged between 2003 and 2007 and between 2007 and 2011 [38]. Between 2003 and 2007 ten countries reported significant decreases and two reported increases, between 2007 and 2011 nine countries reported significant decreases and four countries reported increases. The Nordic countries Iceland, Norway and Sweden, as well as Ireland and the Russian Federation (Moscow), showed decreases most consistent over time. Last month binge drinking was found to have increased in a large number of countries between 2003 and 2007 but showed a similar pattern as seen for last month drinking between 2007 and 2011 (nine report decreases and four report increases). Decreasing trends between 2007 and 2011 in last month cigarette smoking were seen in five countries, and a decreasing trend between 2007 and 2011 in last month cannabis use was seen in four. Taken together, decreasing trends of substance use among Dutch adolescents are not found in general in European countries.

A limitation of our study is that the cross-sectional nature of this study precludes any causal inference. The period between 2005 and 2009 has been a dynamic one, characterised by a major economic crisis, lower family incomes and increasing (job) insecurity. It is hard to attribute the general improvement in health and risk taking behaviour to any particular cause. However, the strong decrease between 2005 and 2009 in alcohol use among adolescents in high school parallels the national campaign that was started in 2006 and used the slogan ‘Not sixteen, not a drop’. The national campaign addressed adolescents and explicitly also parents [18]. Several studies support the idea that interventions addressing adolescents as well as their parents are effective in preventing alcohol use in early adolescents [24–27]. The question is whether the decrease of smoking and cannabis use which parallels the decrease in alcohol is some form of co-occurrence or whether it is due to parallel national anti-smoking prevention programmes, such as minor’s access law or smoking bans in public places [19, 39–42]. Clearly, this study provides evidence for the idea of co-occurrence because the clustering between multiple risk behaviours is strongly consistent over time also in relation to changing trends as was also shown by others [43]. Moreover, on the one hand, the dominancy and importance of targeting alcohol use in adolescents is supported by our results showing that adolescents who do not drink alcohol rarely use other substances and that this group has increased considerably between 2005 and 2009. On the other hand, although several studies showed that smoking and alcohol-drinking are strongly related [6, 43, 44], this and other national and international studies [22, 23, 43, 45] show that there is a group of adolescents who drink without smoking.

Another important finding is that, the campaign to reduce alcohol use, without there being a similarly important campaign to reduce cannabis use, has not lead to the one behaviour being replaced by the other. On the contrary, all unhealthy behaviours went down. Perhaps a common cause has influenced all behaviours simultaneously. For example, teaching parents the importance of influencing their children’s health choices in relation to alcohol might also have influenced the children’s behaviour with regard to cannabis use and smoking. This idea was also addressed in another study showing indeed that parental support and control (via the mediation of rules) were negatively associated with all adolescent risk behaviours (smoking, drinking and cannabis use), but on the other hand, parental rules that specifically targeted alcohol are strongly associated with drinking behaviour but not with smoking and cannabis use [30]. Also the latter study was a cross-sectional study. Longitudinal research is needed to study prevention programmes and the role of parents herewith in order to further investigate whether prevention on alcohol use can be sufficient in preventing not only alcohol use, but other risk behaviours such as tobacco and cannabis use simultaneously. However, it may also be possible that another common cause has influenced all behaviours simultaneously, such as the economic crisis.

## Conclusions

In conclusion, our study shows that health risk behaviours in 2009 1) are lower than in 2005 but still prevalent among young Dutch students (aged 13–16) , 2) are higher at higher age (for all substances), 3) are higher among males (for excessive drinking and cannabis use) and 4) are higher among students with lower educational level (for all substances). Similar risk groups were described in another study among Dutch adolescents [23], except for the relationship between educational level and cannabis use. In the Netherlands, political and social attention for harmful alcohol, smoking and drug use among young people is strong. Future challenges are to further reduce (multiple) risk behaviours and to further postpone the onset of unhealthy habits. A recent European report showed that with respect to the present alcohol use Dutch adolescents are just above the European mean, with respect to cannabis use (last month and age of starting) they are in the top of European countries and with respect to smoking behaviour they have an average position [37]. Recent national policies aim at further postponing the starting age and limiting the availability of substances. Special attention should be given to the youngest age groups and lower educated groups.

## Abbreviations

VMBO: students [lower educational level]
HAVO/VWO: students [higher educational level]
